# An Account of Resuscitation, in a Case of Supposed Death from Yellow Fever

**Published:** 1805-07-01

**Authors:** John Rush

**Affiliations:** Philadelphia


*An Account of Resuscitation, in a Case of supposed Death from Yellow Fever; by* JOHN RUSH, M. D. *of Philadelphia.*

IN the month of August of the year 1798, the yellow fever appeared in Marcus Hook, a small village situated on the banks of the river Delaware. The crews of the United States vessels Ganges and Retaliation, which lay along-side of the village, were at the same time attacked with this disease. Upon the first indisposition, each person was removed from the vessels and conveyed to tents fitted up for his reception, on an elevated and healthy spot of ground in the neighbourhood; where, through the humane attentions of his commander, he was provided with every necessary that his situation required.

Out of sixty seamen, ordinary seamen, and marines, who had been sent to the tents, many had the disease mildly, some suffered severely, and four died, on the third, fifth, and seventh days, with black vomiting and other symptoms of great malignity. Of the whole number, I have selected a case, which, from the rareness of its occurrence, may be interesting and important.

The particular symptoms that marked the progress of the disease in this case, from the apparent cessation of life to its complete resuscitation, I am unable to state, owing to the circumstance of the daily journal remaining in the possession of my assistant Mr. Parker, after he had retired from the United States' service. What I observed at the time of my visits, which through necessity were short and few, I have correctly stated, and have no reason for doubting the truth of what is farther advanced, from the representation of Mr. Parker.

CASE.

James Clark, an ordinary seaman belonging to the Ganges, about nineteen years of age and of a hale constitution, was attacked on the seventh of September with the yellow fever. The symptoms were such as characterize the malignant forms of this disease. The force of the disease seemed principally exerted on the arterial system, while the muscular and nervous systems appeared to be but secondarily affected. The pulse was depressed at the commencement of the attack, but rose soon afterwards, and became full and strong. Twenty-four oz. of blood, in all, were taken from his arm in the first paroxysm; during which, he was copiously purged with strong doses of calomel. On the second day bleeding and purging were discontinued, and mercurial frictions, together with small and repeated doses of calomel, were prescribed, in order to produce a salivation. This, however, could not be effected. The disease, notwithstanding the use of a variety of stimulants, such as brandy, ether, and laudanum, arrived at the last stage, when, on the morning of the fourth day, the black vomiting began and continued until twelve o'clock at noon; at which time, it was said, he had expired. Upon paying my second visit to the tents, at four o'clock in the afternoon of the same day, I saw the body of Clark lying in a coffin and apparently lifeless. On closely examining it, I observed the pale yellow, that previously tinged tinged the temples, nails and neck, changed to an orange-like hue, and interspersed with purplish spots resembling petechiæ: neither pulse nor heat were perceptible, nor was respiration discoverable on the mirror, which was held before the mouth. Putrefaction, however, had not taken place; the lower jaw was still flexible; and upon a more minute examination, I felt (or thought I felt,) a slight warmth about the epigastric region. With such slender and evanescent symptoms of life, experiment indeed promised little. But something I was resolved to attempt: I therefore ordered the body to be covered with warm ashes from the cook's fire, and a gill of very strong brandy toddy to be poured down the throat every half hour. Being called away, I could not wait to see the effect of these remedies; but requested Mr. Parker to continue the use of them, whilst any hope remained of their being successful. On my return at sunrise the following morning, I had the pleasure to find Clark propped up, indulging himself with soup. From Mr. Parker I learned, that about eight o'clock, after he had received a quart of brandy, he began to respire; that the brandy was continued in the same proportion which I had prescribed, until eleven o'clock, when he was so far recovered, as to complain of the warmth of the ashes? that he was then taken out of the coffin, and laid on straw on the ground. Port wine sangree was then substituted for brandy, and was regularly administered until day light, when he refused to take any more, and called for food.

On the treatment of the above case, it may be proper to remark, that had convulsions or spasms attended the apparent dissolution, I should have hesitated in pouring a fluid down the throat; as, when death occurs in convulsions, the glottis might not be completely closed, while the muscles of the epiglottis, partaking also of the general convulsion, might retain it in an erect position: hence a fluid would pass into the lungs, as well as into the stomach, a circumstance which would prevent resuscitation. But in cases like Clark's, where muscular relaxation accompanied the apparent extinction of life, the epiglottis must necessarily be in contact with the glottis, and thereby prevent the admission of a fluid into the lungs.

If the history of the above case should serve to prevent premature interment, and lead to the use of remedies for resuscitation in doubtful cases of death from fever, as well as from causes which induce it suddenly, it will be to me a high gratification.

*Aug.* 17, 1804.

